# Effect of green tea extract antioxidant on dentin shear bond strength and resin-tag penetration depth after non-vital bleaching

**DOI:** 10.12688/f1000research.133313.2

**Published:** 2023-09-08

**Authors:** Darin Safinaz, Paramita Widyandari, Ratna Meidyawati, Citra Kusumasari, Dewa Ayu Nyoman Putri Artiningsih

**Affiliations:** 1 Department of Conservative Dentistry, Faculty of Dentistry, University of Indonesia, Depok, West Java, 16424, Indonesia

**Keywords:** Tooth bleaching, green tea extract, antioxidant

## Abstract

**Background:** 35% Hydrogen peroxide (H _2_O _2_) as an active material for internal bleaching can produce free radicals that can affect resin tag penetration into the dentinal tubules. Application of 10% and 35% green tea (GT) extract as an antioxidant after 2 minutes are expected to remove free radical residues and increase dentin shear bond strength and resin tags penetration depth after non-vital bleaching.

**Methods:** 30 extracted healthy human premolars were cut horizontally 2 mm from the Cemento Enamel Junction margin to the crown part, then cut in a mesio-distal direction into two parts. The specimens were divided into five groups: normal dentin, post bleaching dentin, delayed 2 weeks, 10% GT, and 35% GT group. Non-vital walking bleach with 35% H _2_O _2_ gel was done to all groups except control group. Soon after, 10% and 35% GT extract gel were applied on dentin for 2 minutes, then the specimens were rinsed-off with aquabidest for 2 minutes and dried. All specimens were etched and bonded with an etch-and-rinse adhesive system and filled with resin composite. The shear bond strength assessment was carried out using a Universal Testing Machine (UTM) with a cross-head speed of 0.5 mm/minute. Confocal laser scanning microscopy (CLSM) with a wavelength of 560 nm and a lens magnification of 40x was used to analyze the resin tag penetration. Data were analyzed by one way ANOVA and t-test.

**Results:** There was a significant difference in resin tag penetration depth and shear bond strength between applying 10% and 35% GT extract (p < 0.05). The 35% GT extract group resulted in a significantly longer resin tag penetration than the 10% GT extract group.

**Conclusions:** The application of 35% GT extract is more effective than 10% GT extract as an antioxidant for increasing the shear bond strength of composite resin after internal bleaching.

## Introduction

35% hydrogen peroxide (H _2_O _2_) is commonly used as an active material for non-vital bleaching; however, it can produce radical oxygen species (ROS). ^1^^,^^2^ These ROS can form bubbles trapped inside the dentinal tubules interfering with resin tag penetration into the dentinal tubules. ^3^ Moreover, they can also inhibit the polymerization process due to the disrupting of the vinyl radical group propagation process in the composite resin. So, the termination process occurs earlier in the formation of polymer chains. ^4^

The inhibition of the resin tag penetration and polymerization process can reduce physical properties such as the bond strength of adhesive material to dentin, and increase the microleakage of composite resin restorations. ^4^ Previous studies have stated that naturally, free radicals in the dentinal tubules take one to three weeks to disappear. ^5^ However, delayed restoration can lead to a higher risk of crown fracture and loss of temporary restorations, leading to re-discoloration. Free radicals from the bleaching process can be eliminated by applying antioxidants. ^6^^,^^7^ Recently, natural antioxidants from green tea extracts are being developed to eliminate free radicals after bleaching. ^8^ The concentration and time of application of antioxidant agents have major roles in enhancing their antioxidant effects. ^9^

Applying of 10% green tea (10% GT) extract for 10 minutes effectively increased shear bond strength and reduced microleakage formation, which is closely related to the ability of the resin tag penetration which can affect the retention of the composite resin restoration to dentin. ^10^^–^^12^ The concentration of antioxidants must be proportional to the concentration of hydrogen peroxide, and the application of 35% sodium ascorbate for 2 minutes has been proven to remove free radicals after the application of 35% H _2_O _2_. ^13^^,^^14^ Therefore, 35% green tea (35% GT) extract was used in this study, and it was expected that it could remove free radical residue and increase the resin tag penetration depth. ^13^

Based on our knowledge, there is no study that has investigated the effects of application of 2 minutes 35% GT extract on the shear bond strength and penetration of resin tags on dentin after non-vital bleaching with 35% H _2_O _2_. Thus, this study aims to evaluate the effect of green tea extract application on the shear bond strength and penetration of resin tags on dentin after non-vital bleaching. The null hypothesis was that there would be no difference on the shear bond strength and penetration of resin tags on dentin after non-vital bleaching.

## Methods

### Specimen preparation

This study was held on March - April 2022. Due to there being two components of the study (sheer bond strength and resin tag penetration), two ethics applications were submitted. The study was approved by the Ethics Committee of Universitas Indonesia (10/Ethical Approval/FKGUI/III/2022 approved on March 9 ^th^ 2022) and deemed exempt by the Ethics Committee of Universitas Indonesia (09/EthicalExempted/FKGUI/IV/2022, on March 11 ^th^ 2022). It was conducted in accordance with the Declaration of Helsinki. 30 maxilla premolars, previously extracted for orthodontic reasons, which were free of caries, fractures, and defects were included in this study. The teeth were soaked in a thymol solution (0.1%; pH 7.0) for 1 week post extraction. Then, after 1 week the teeth were placed in 4 ^o^C distilled water, and have to be used within 1 month post extraction. ^15^ The root portion of each tooth was removed 2 mm below the cement-enamel junction with a double side disc diamond bur (Buehler, Lake Bluff, IL, USA). The coronal portion was sectioned mesiodistally, and the buccal and palatal portions were used. Each portion is considered to be one specimen (n=60). 55 were used in total during this research.

The dentin surfaces of the specimens were flattened using 600- and 1200-grit sandpaper and polished with felt discs (Arotec, Cotia, SP, Brazil) impregnated with alumina paste (0.5 μm) until a specimen thickness of 3 mm was obtain. The specimens were washed ultrasonically in distilled water for 5 minutes to eliminate any residue. For the shear bond strength test the specimens were fixed in self-cure acrylic 20 mm, and for the resin tag penetration test the specimens were fixed in plasticine 1×1 mm. A mould with a diameter of 2 mm was glued over the specimen in the pulp chamber using plasticine.

The specimens were randomly divided into 5 groups (for resin tag penetration test n=6, for shear bond strength test n=5). Group 1: Non-bleached without green tea extract (normal dentin/negative control group). Group 2: Bleached without green tea extract (post bleaching dentin/positive control group). Group 3: Bleached without green tea extract and delay for 2 weeks before being restored (delay 2 weeks). Group 4: Bleached+10% Green tea extract for 2 min (10% GT), Group 5: Bleached+35% Green tea extract for 2 min (35% GT).

### Green tea extract preparation

Green tea extract with a concentration of 10% and 35% was made by grinding 1 kilogram of pure green tea leaves (Tea Heaven, Indonesia) into a powder. The green tea powder was then soaked in 3 litres of ethanol and left for 24 hours. The mixture was then concentrated using an evaporator. To change the consistency into a 35% gel, 35 grams of extract were added to 65 grams of water and mixed with 2.5 grams of carboxymethyl cellulose (CMC). To change the consistency into a 10% gel, 10 grams of green tea extract were added to 90 grams of water, then mixed with 2.5 grams of CMC. Then the 10 % and 35 % extracts were neutralized by adding triethanolamine drop by drop until the gel reached a pH of 7. This was determined by using a laboratory digital pH meter (Transinstrument BP3001). The extract was placed in a clean container and the probe was dipped into the container. The pH meter started to calculate the pH; once the pH number on the screen remained steady, that was the pH number. The probe was then removed and cleaned and added back, as more triethanolamine was gradually added and the process was repeated until a pH of 7 was reached. Green tea extract was tested by spectrophotometry before and after mixing the gelling ingredients, to determine the flavonoid levels did not change.

### Internal bleaching procedure

The walking bleach procedure was carried out on specimens of groups 2, 3, 4, and 5 by applying 1 mm thick of 35% H _2_O _2_ gel bleaching agents (Opalescence Endo, Ultradent, USA) into the mould that has been placed on the pulp chamber. The moulds were covered with plastic wrap and then the specimens were stored in an incubator for 5 days at 37 ^o^C and 100% relative humidity. The cavity was cleaned with distilled water for 1 minute, then dried with a three-way syringe. At the end of each day of treatment, the bleaching gel was rinsed with distilled water for 1 minutes.

### Green tea extract application procedure

The 10% green tea extract gel was applied in the mould for 2 minutes for group 4, and 35% GT extract gel was applied in the mould for 2 minutes for group 5. Then, the cavity was cleaned with distilled water for 2 minutes and dried using a three-way syringe. Meanwhile, in groups 1, 2, and 3, no antioxidants were given.

### Bonding and restoration procedure

All specimens in all test groups were etched with 37% phosphoric acid (Scotchbond, 3M ESPE) for 10 seconds and then rinsed and partially dried to moist conditions and to prevent collagen collapse. After that, for the resin tag penetration test the adhesive was mixed with 0.1% rhodamine B isothiacyanate (RITC) fluorescent dye (Aldrich Chem. Co., Milwaukee, WI, USA) and for shear bond strength test the adhesive was not mixed with anything. Then, the adhesive (Adper Single Bond 2, 3M ESPE, USA) was applied using a micro brush to the specimen and allowed to stand for 20 seconds, then air-dried using a three-way syringe for 5 seconds and light cured with a light curing unit (DBA, Guilin Woodpecker Medical Instrument Co., China, wave length 440-490 nm, dan light intensity 1200 mW/cm ^2^) for 10 seconds. For the shear bond strength test, the specimen was restored with composite resin (Filtek Z350XT, 3M ESPE, USA).

### Confocal laser scanning microscope (CLSM) observation

The resin tag penetration in the specimen can be seen from the luminescence of rhodamine B through CLSM (LSM 700, Carl Zeiss Microscopy, Germany) with a wavelength of 560 nm and a lens magnification of 40x. The depth of penetration of the resin tag into the dentinal tubules was observed by two experienced observers at the same time and the mean fluorescence intensity (MFI) was calculated using the ZEN 2010 software (Carl Zeiss Microscopy GmbH, Jena, Germany).

### Shear bond strength test

The shear bond strength assessment was carried out using a Universal Testing Machine (AG 5000E, Shimadzu) with a cross head speed of 0.5 mm/minute. The load applied when restoration detached from specimen is recorded. The value of shear bond strength was calculated and presented in megapascal (MPa). ^15^

### Statistical analysis

The data was analyzed using one-way ANOVA, followed by post-hoc Tamhane test for resin tag penetration and post-hoc Bonferroni test for shear bond strength test (IBM SPSS Statistics 24 version program, IBM Corp., Armonk, NY, USA).

## Results

Table 1 shows that the longest penetration of resin tag is in the 35% GT group with a mean value of 190.1 (SD 33.6) μm. Meanwhile, the post-bleaching dentin group showed the shortest resin tag penetration with a mean value of 87.4 (SD 6.9) μm. The results of the one-way ANOVA test showed a value of 0.001 (p<0.05), which means that there was a significant difference in the depth of penetration of the resin tags from the five groups.

**Table 1.** Mean, standard deviation, and significancy between group of resin tag penetration (μm).

Treatment	Number of specimens	Mean (SD)	p-value
Normal dentin	6	146.9 (10.1) ^x^	**0.001** *
Post-bleaching dentin	6	87.4 (6.9) ^y^	
Delay 2 weeks	6	134.4 (9.5)	
10% GT	6	107.2 (18.9) ^x,z^	
35% GT	6	190.1(33.6) ^y,z^	

GT = Green Tea; x = significance difference normal dentin with 10% GT; y = significance difference post bleaching dentin with 35% GT; z = significance difference 10% GT with 35% GT.

* One-way ANOVA test with p<0.05.

Meanwhile, to determine the difference in depth of resin tag penetration between treatment groups with one another, the post-hoc Tamhane test was conducted ( Table 1). The results of the post-hoc test in Table 1 show that there was a significant difference in the resin tag penetration between the 10% GT group and the 35% GT group (p=0.008). The 35% GT group showed a longer depth of resin tag penetration compared to the 10% GT group. There was also a significant difference of resin tag penetration between the 10% GT group and the normal dentin group. The 10% GT group showed a shorter depth of resin tag penetration than the normal dentin. There was no significant difference of resin tag penetration between the 35% GT group and the normal dentin group.

**Figure 1.**
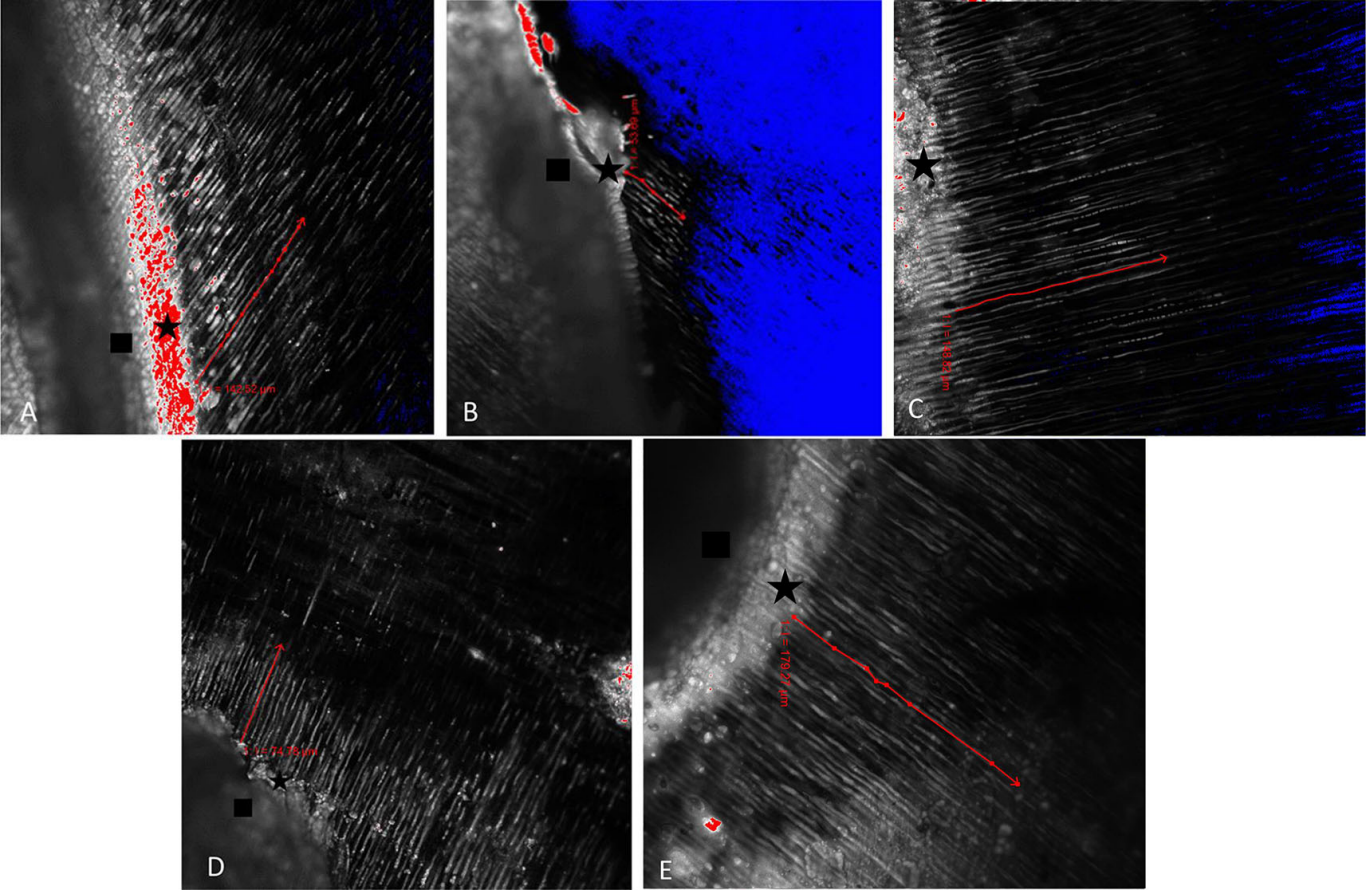
The red arrow indicates the depth of penetration of the resin tag on (A) Normal dentin group, (B) Post bleaching group, (C) Delay 2 weeks group, (D) 10% GT group, (E) 35% GT group. Rectangle indicates the adhesive; star indicates the hybrid layer; blue area means there is no adhesive penetration/no fluorescence, red area means the thickest fluorescence.

The results of CLSM imaging of the resin tag penetration for each group can be seen in Figure 1A, B, C, D, and E. From the picture, it can be seen that the longest resin tag penetration is in the 35% GT group. In addition to being longer, the resin tags formed in the 35% GT group seemed more abundant, thicker, and continuous than the other group. The results of the shear bond strength test between groups are described in Table 2. The data obtained showed that the value of shear bond strength in the 35% GT group had the highest mean value, which was 10.84 (SD 2.68) MPa. The lowest shear bond strength value was in the post bleaching dentin group, which was 3.52 (SD 0.22) MPa. The results of the one-way ANOVA test showed significantly different values from each treatment group, as seen from the p-value of 0.001 with p<0.05.

**Table 2.** Mean, standard deviation, and significancy between group of shear bond strength (MPa).

Treament group	Number of specimens	Mean (SD)	p-value *
Normal dentin	5	10.14 (1.85) ^a^	**0.001** *
Post-bleaching dentin	5	**3.52 (0.22)** ^**b**^	
Delay 2 weeks	5	8.65 (2.09)	
10% GT	5	5.42 (0.54) ^a,c^	
35% GT	5	**10.84 (2.68)** ^**b,c**^	

GT = Green Tea; a = significance difference normal dentin with 10% GT; b = sigficance difference post bleaching dentin with 35% GT; c = significance difference 10% GT with 35% GT.

* One-way Anova test with p<0.05.

To determine the difference in shear bond strength between treatment groups, the results of the post-hoc Bonferroni test are displayed in Table 2. The results show a significant difference in the shear bond strength between the 10% GT group and the 35% GT group (p=0.001). The 35% GT group showed the higher shear bond strength compared to 10% GT group. These results show that the application of 35% GT extract can increase the post-bleaching shear bond strength more than the application of 10% GT. The 10% GT group showed lower shear bond strength than normal dentin. There was no significant difference of shear bond strength between the 35% GT group and the normal dentin group.

## Discussion

H _2_O _2_ is a strong oxidizer that has a faster reaction and is effective in whitening teeth. These reactive molecules break the long chains of the chromophore molecules, making them diffuse more and become colourless. ^16^ However, the active ingredient of bleaching H _2_O _2_ will release large amounts of free radicals, so it can produce a negative effect in the form of reducing the bonding of the resin material with dentin and enamel. The oxygen formed can interfere with the penetration of the adhesive material so that the resin tag becomes sparse, short, not well-defined, and structurally incomplete, and in some areas the resin tag is not formed at all. ^17^ This situation results in decreased bond strength due to disturbances in the polymerization process, and changes in the chemical structure of enamel and dentin. ^18^^–^^20^

To eliminate the negative effects of these free radicals, Torres *et al*. (2006) and Ismail *et al*. (2017) stated that the antioxidant agents are oxidation inhibitors. They can neutralize free radicals by binding free radicals through hydrogen atom donation or electron transfer so that free radicals become more stable, less reactive, and less dangerous. Thus, restoration procedures can be done immediately after the bleaching procedure. ^21^^,^^22^ This study uses green tea as an antioxidant agent because it is a natural antioxidant agent and biocompatible. The toxicity of natural ingredients is not always linearly proportional to their concentration so an increase in the concentration of a substance does not always increase its toxicity. ^8^

The content of flavonoids contained in green tea is influenced by weather, climate, tea varieties, geographical location, soil conditions, leaf age, and the way of picking. ^23^ The green tea used in this research had an unfermented processing process where there is inactivation of the polyphenol oxidase enzyme present in the fresh tea shoots, so that it is not oxidized much and the content of polyphenols or catechins, especially EGCG, is higher with the main nutrients being maintained. ^23^ El-Hack *et al*. (2020) stated that EGCG in green tea is 100 times more effective in eliminating free radicals than vitamin C and 25 times more effective than vitamin E. ^24^

The results of this study showed that the longest penetration of resin tags and the highest number of shear bond strength was in the 35% GT group ( Tables 1 and 2). From the post-hoc test, it appears that there is a significant difference in the resin tag penetration and shear bond strength between the 35% GT group and the post-bleaching dentin group (p<0.05). In the 35% GT group, there was a longer resin tag formation ( Figure 1B & E) and higher shear bond strength compared to the post-bleaching dentin group. These results are in line with research conducted by Briso *et al*. (2012) which reported that the use of antioxidants immediately after bleaching can reduce the presence of reactive oxygen in post-bleaching tooth tissue. ^25^ One theory is that hydrogen peroxide can split into water and oxygen in the collagen matrix and dentinal tubules. Release of oxygen can interfere with resin penetration into dentin which has been etched. ^26^ Gündoğdu and Yılmaz (2020) stated that the strong antioxidant activity of green tea has been attributed to its high content of catechins and flavanols, which can neutralize free radicals by donating hydrogen from the hydroxyl group in its structure. ^11^

There was no significant difference in resin tag penetration and shear bond strength between the 35% GT group, the normal dentin group, and the two-week delayed group (p>0.05) ( Tables 1 and 2). These results are in line with the findings of a study conducted by Freire *et al*. (2009) who state that the concentration of antioxidants must be directly proportional to the concentration of hydrogen peroxide. ^13^ In other words, the concentration of 35% green tea extract is effective in eliminating 35% H _2_O _2_ free radicals. However, the average penetration value of the resin tag in the 35% GT group was longer than the normal dentin group; this might be because, after the internal bleaching procedure with 35% acidic H _2_O _2_ (pH 5), there was a change in the organic and inorganic components of dentin which causes an increase in the diameter of the dentinal tubules. ^8^

Although the administration of 35% GT extract antioxidants can induce cross-linking of dentin collagen molecules, induce the remineralization process, and inhibit matrix metalloproteinase (MMP) activity, only 2 minutes of application may not be able to form collagen and minerals perfectly as in normal dentin, so that tubule diameter will still be larger than normal dentin. Dentinal permeability is closely related to the functional diameter of the dentinal tubules, so the larger the functional diameter, the higher the flow rate of liquid that can penetrate to the dentinal tubule. This liquid can be assumed as a bonding material; the bonding used is Adper Single Bond 2 (3M, USA) which contains spherical silica filler particles with a diameter of five nanometers. The small size causes Adper Single Bond 2 to have a better ability to penetrate into dentinal tubules, as its small particles are stable in the form of a colloidal suspension, which means that they do not combine with one another and do not agglomerate. This allows the Adper Single Bond 2 to have the ability to penetrate into deep dentinal tubules. ^8^

In addition, the results of the post-hoc test also showed that there was a significant difference in the penetration depth of the resin tag and shear bond strength between the 10% GT group and the 35% GT group (p<0.05) ( Tables 1 and 2). The 35% GT group showed significantly longer resin tag formation than the 10% GT group ( Figure 1D & E). Similarly, the value of shear bond strength in the 35% GT group was significantly higher compared to the 10% GT. The result is in line with research conducted by Freire *et al.* (2009) who revealed that the concentration of antioxidants had a greater effect than the exposure time on the rate of free radical reduction. ^13^ In addition, Hamid *et al.* (2010) showed that when higher concentrations of antioxidants were used, the time required to eliminate all free radical residues was reduced. ^27^

Although previous studies have stated that the use of 10% GT extract for 10 minutes is effective as an antioxidant agent after bleaching, its use for only two minutes appears to be less effective in eliminating free radicals. It can be seen from the results of the post-hoc test that there was no significant difference in the depth of penetration of the resin tag and shear bond strength between the 10% GT group and the post-bleaching dentin group (p<0.05) ( Tables 1 and 2). However, there was a significant difference between the 10% GT group and the normal dentin group (p>0.05).

In this study, we only use one type of adhesive where the particle size of the adhesive material affected the penetration ability of the resin tag into the dentinal tubules. So further research is needed by comparing the penetration of resin tags from several types of adhesives. The results of the CLSM analysis could not see whether the residual oxygen in the dentinal tubules after internal bleaching had been eliminated after the application of green tea antioxidants, so further research using SEM is needed to be able to see visually whether the oxygen bubbles have completely disappeared in the dentin after internal bleaching after the application of green tea antioxidants. Therefore, the null hypothesis which stated that there was no difference in the effect of the application of green tea extract antioxidant with different concentrations for 2 minutes on dentin shear bond strength and resin-tag penetration depth after internal bleaching was rejected.

## Conclusion

The application of 35% GT extract antioxidant for 2 minutes increased the shear bond strength and resin tag penetration, compared to the application of 10% GT extract for 2 minutes on dentin after internal bleaching using 35% H _2_O _2_.

## Data Availability

figshare: Resin tag and shear bond strength data, https://doi.org/10.6084/m9.figshare.22568338.v1. ^28^ This project contains the following underlying data:
- Shear bond strength dataset - Resin tag penetration data -
Figures for the manuscript -
Figure data figshare: Resin Tag Microscopic images, https://doi.org/10.6084/m9.figshare.22761662.v1. ^29^ This project contains the original microscopy images. figshare: Effect of Green Tea Extract Antioxidant on Dentin Shear Bond Strength and Resin-Tag Penetration Depth after Non-vital Bleaching, https://doi.org/10.6084/m9.figshare.22568581.v3. ^30^ Data are available under the terms of the Creative Commons Zero “No rights reserved” data waiver (CC0 1.0 Public domain dedication).
