# Supplementary material for: Effect of green tea extract antioxidant on dentin shear bond strength and resin-tag penetration depth after non-vital bleaching
Source: F1000Res. 2023 Sep 8;12:660. Originally published 2023 Jun 13. [Version 2] doi: 10.12688/f1000research.133313.2 (PMC11319902; doi:10.12688/f1000research.133313.2)
Supplement: Peer review history

# Reviewer report: Reviewer response for version 2

**DOI:** 10.5256/f1000research.155025.r310515

**Published:** 2024-08-12

**Authors:** Henrico Badaoui Strazzi Sahyon

**Affiliations:** 1 Department of Prosthodontics and Periodontology, Bauru School of Dentistry, University of Sao Paulo (USP), Bauru, SP, Brazil

Manuscript: Effect of green tea extract antioxidant on dentin shear bond strength and resin-tag penetration depth after non-vital bleaching. Dear authors, it was a pleasure to review your article paper. My comments are mentioned below. 

 The present in vitro study aimed to evaluate the effect of 10% and 35% green tea extract after 2 minutes of application on dentin shear bond strength and resin tags penetration depth after non-vital bleaching. Some comments about the manuscript are described below. 

**Abstract**

 1) Use in vitro study.

 2) Use the sample size per group.

 3) The abstract did not mention the time of action of the bleaching agent and the number of sections.

 4) Were the dentin samples etched with which material? Which adhesive was used?

 5) Sample size per analysis.

 6) Results section can be improved and insert more details of the data and P values. 

 7) The conclusion should emphasize the broader implications and significance of the study’s findings, rather than merely reiterating the results.

 8) Insert more keywords in relation to the analyses evaluated.

 9) In general, the abstract in poorly described and must be improved. 

**Introduction**

 1) The authors claim: “The concentration of antioxidants must be proportional to the concentration of hydrogen peroxide, and the application of 35% sodium ascorbate for 2 minutes has been proven to remove free radicals after the application of 35% H₂O₂.” Why did the authors not include a group using a more common and well-evaluated antioxidant in the literature, such as sodium ascorbate or ascorbic acid? The efficiency of green tea should be evaluated and compared to other antioxidants at the same concentration.  

 2) The null hypothesis is incomplete. “The null hypothesis was that there would be no difference on the shear bond strength and penetration of resin tags on dentin after non-vital bleaching.” Compared to what? Must be compared to something. 

 3) The authors incorporated a group performing adhesion after 2 weeks. Therefore, the authors should include two additional groups: the first one performing bleaching and using a 10% antioxidant, then performing adhesion after 2 weeks; and the second one performing bleaching and using a 35% antioxidant. Furthermore, the authors must evaluate the influence of the antioxidant on unbleached dentin to assess the isolated effect of the antioxidant. Consequently, the design of the study was not well executed.  

**Materials and Methods**

 1) How did the authors measure the thickness of the bleaching agent? How was this standardization performed?

 2) The authors did not specify the time and duration of the beaching treatment. 

 3) How the authors applied the antioxidant? There was not a standardization of the amount used for each sample. Should be used the same amount. Furthermore, why did the authors adopt 2 minutes of action of the antioxidant? References and reasons for that must be cited. 

 4) “the specimen was restored with composite resin” What was the thickness of the restoration?

 5) More details of the CLSM are required. 

 6) Was a macro shear bond strength methodology used? This is confusing. More information about the test is required. Five samples per group for macro shear bond strength analysis is low. The authors should consider increasing the sample size for this analysis. 

 7) Why did the authors not present the adhesive failures? Cohesive, mixed, and adhesive? This information is crucial for bonding tests. 

 8) Scanning electron microscope of the failures are required too.

 9) Why did the authors not evaluate the thermal aging process? Aging is crucial for bonding tests. The authors did not assess whether the aging process could affect the antioxidants, bleaching, and bonding efficiency after aging. This information is very important for understanding the long-term effects and reliability of the treatments.

 10) Was the resin tag penetration analysis evaluated by the inter-examiner Kappa test? Where is the concordance agreement between the examinators? Was used a power test to obtain the sample size of the analyses?  

 11) The statistical information is incomplete. Must contain about the normality and homogeneity tests too.

 12) A table containing the materials details (composition, batch number and other) should be inserted. 

 13) A schematic figure of the methodology would be beneficial to facilitate the readers' comprehension. 

 14) The authors should not consider the “normal dentin” as a negative control once this is the best scenario. The negative control should be the group 2 submitted to bleaching agent, once it is obvious that the bleaching treatment can induces some deleterious effects on the dental substrate.  

 15) The design of the study must be deeply reconsidered. 

**Results**

 1)       Tables 1 and 2 are confusing. Why did the authors not compare the “Delay 2 weeks” group with the other experimental groups? I suggest using letters to compare all the groups and employing different letters to indicate statistically significant differences among them.

 2)   The authors should consider including failure modes and SEM evaluation to corroborate the shear bond strength analysis. 

**Discussion**

 1) Please, insert the full meaning of EGCG.

 2) An important concern: Does the application of green tea extract promote color alteration of the substrate? Furthermore, could this application during the bleaching treatment could be exacerbated? Did the authors evaluate these parameters using color stability analysis and whiteness stability too?

 3) Did the authors confirm the real effectives of green tea extract on reducing the concentration of oxygen peroxide? More information on the discussion is necessary. 

**References**

 1) The authors should incorporate more up-to-date references related to the study. I suggest three recent studies that evaluated similar factors in dental bleaching using antioxidant agents. These references can corroborate the present study. The references are: Ref [1,2,3]

 In general, the present study presents some interesting data. However, this investigation has significant flaws in its design and methodology. Additionally, the English language needs to be revised and improved in some sentences. The authors need to address the comments above and enhance the manuscript content for it to be reconsidered. Therefore, the manuscript cannot be accepted in its current form and requires substantial improvements.

Is the work clearly and accurately presented and does it cite the current literature?

Partly

If applicable, is the statistical analysis and its interpretation appropriate?

Partly

Are all the source data underlying the results available to ensure full reproducibility?

Partly

Is the study design appropriate and is the work technically sound?

No

Are sufficient details of methods and analysis provided to allow replication by others?

No

Reviewer Expertise:

Dental Materials, Restorative Dentistry, Prosthodontics, and Implantology.

I confirm that I have read this submission and believe that I have an appropriate level of expertise to confirm that it is of an acceptable scientific standard, however I have significant reservations, as outlined above.

## References

[1] : In vitro study on how antioxidant solutions affect enamel surface characteristics and bonding interface of ceramic laminate veneers luting after dental bleaching. *J Mech Behav Biomed Mater* .2022;133: 10.1016/j.jmbbm.2022.105322 105322 10.1016/j.jmbbm.2022.105322 35724532

[2] : Does the use of antioxidant agents after dental bleaching compromise the aesthetic results of ceramic laminate veneers?. *J Mech Behav Biomed Mater* .2023;145: 10.1016/j.jmbbm.2023.106027 106027 10.1016/j.jmbbm.2023.106027 37487466

[3] : Effect of Antioxidant Agents After Dental Bleaching on Color Stability and Mechanical Properties of Bonding Interface Components in Ceramic Laminate Veneer Luting. *Oper Dent* .2023;48(4) : 10.2341/21-200-L 391-403 10.2341/21-200-L 37302146

# Reviewer report: Reviewer response for version 2

**DOI:** 10.5256/f1000research.155025.r300338

**Published:** 2024-07-15

**Authors:** Diatri Nari Ratih

**Affiliations:** 1 Universitas Gadjah Mada, Yogyakarta, Special Region of Yogyakarta, Indonesia

1. Keywords: It  needed more keywords, such as shear bond strength and resin tag penetration

 2. Introduction: 

- There is no explanation why the authors chose 10% GT for comparison with 35% GT.
- There is no explanation why the authors chose 2 minutes for application.

3. Methods: 

- Is there any Kappa test to calibrate between 2 observers?
- There is no explanation for determining the sample size.

4. Discussion:

- How do we know the EGCG content in GT that the authors used is high since the content of GT is not only EGCG?
- It is stated in the article that the average penetration value of the resin tag in the 35% GT group was longer than the normal dentin group; this might be because, after the internal bleaching procedure with 35% acidic H2O2 (pH 5), there was a change in the organic and inorganic components of dentin which causes an increase in the diameter of the dentinal tubules. This statement is contradictory because normal dentin (negative control) was not applied to bleaching.

Is the work clearly and accurately presented and does it cite the current literature?

Partly

If applicable, is the statistical analysis and its interpretation appropriate?

Partly

Are all the source data underlying the results available to ensure full reproducibility?

Partly

Is the study design appropriate and is the work technically sound?

Yes

Are the conclusions drawn adequately supported by the results?

Yes

Are sufficient details of methods and analysis provided to allow replication by others?

Partly

Reviewer Expertise:

Endodontic

I confirm that I have read this submission and believe that I have an appropriate level of expertise to confirm that it is of an acceptable scientific standard, however I have significant reservations, as outlined above.

# Reviewer report: Reviewer response for version 2

**DOI:** 10.5256/f1000research.155025.r204956

**Published:** 2023-09-18

**Authors:** Myrna Zakaria

**Affiliations:** 1 Department of Restorative Dentistry, Faculty of Dentistry, Universiti Malaya, Kuala Lumpur, Federal Territory of Kuala Lumpur, Malaysia

-

Is the work clearly and accurately presented and does it cite the current literature?

Partly

If applicable, is the statistical analysis and its interpretation appropriate?

Partly

Are all the source data underlying the results available to ensure full reproducibility?

Partly

Is the study design appropriate and is the work technically sound?

Partly

Are the conclusions drawn adequately supported by the results?

Yes

Are sufficient details of methods and analysis provided to allow replication by others?

Partly

Reviewer Expertise:

Endodontics, oral microbiology, dental materials

I confirm that I have read this submission and believe that I have an appropriate level of expertise to confirm that it is of an acceptable scientific standard.

# Reviewer report: Reviewer response for version 1

**DOI:** 10.5256/f1000research.146292.r179218

**Published:** 2023-07-03

**Authors:** Myrna Zakaria

**Affiliations:** 1 Department of Restorative Dentistry, Faculty of Dentistry, Universiti Malaya, Kuala Lumpur, Federal Territory of Kuala Lumpur, Malaysia

The study highlights the effect of anti oxidants (green tea extract) in improving bond strength of adhesives after external bleaching. The study evaluated the bond strength supported by CLSM evaluation to observe the penetration depth of the adhesive. However, I personally think that the CLSM images is unclear, therefore interpretation is rather dubious, particularly to being asses quantitatively. 

 In details, here are my concern to be addressed:

**Abstract:**

- Please mention the aim of the study in the abstract.
- Please specify the method in abstract section including the statistics. What was the post hoc analysis following the ANOVA and what was the t test for? (there was no t test mention in the script)?
- Please change 'aquabidest' to 'distilled water'.
- Results is unclear, it seemed that author only explained comparison between 2 groups (leads to assumption the result of t test, but then it was not in the methods and results of the manuscript).
- Please specify the results of shear bond strength of the 5 groups, then mention the significance. Since author is not only comparing 10% and 35% GT extract, how was the results for the other groups tested (highest? Lowest?)? Was there any host hoc analysis done?

**Introduction:**

- What was the rationale in using 10 and 35% extract? Generalizing the results from 35% sodium ascorbate to this study is irrelevant. GT extract differs from sodium ascorbate, therefore it cannot be used as a standard for concentration.
- Introduction paragraph 3:
  “The concentration of antioxidants must be proportional to the concentration of hydrogen peroxide, and **the application of 35% sodium ascorbate for 2 minutes has been proven to remove free radicals after the application of 35% H** **2O _2_.** ^13^ Therefore, 35% green tea (35% GT) extract was used in this study, and it was expected that it could remove free radical residue and increase the resin tag penetration depth. ^13^ **The application of 35% sodium ascorbate for 2 minutes has previously been shown to be effective in removing residual free radicals after application of 35% H** **2O _2_**. ^14”^ - Even though they have different citations, the two phrases have the same meaning. Avoid overwriting it.
- What was the colour of the green tea extract? Isn’t there a chance of staining to the tooth compromising the bleaching results?

**Methods:**

- "The dentin surfaces of the specimens were flattened using 600- and 1200-grit sandpaper and polished with felt discs (Arotec, Cotia, SP, Brazil) impregnated with alumina paste (0.5 μm)." - what was the final specimen dimension?
- "The specimens were washed ultrasonically in distilled water for 5 minutes to eliminate any residue. For the shear bond strength test the **specimens were fixed in self-cure acrylic 20 mm**, and for the resin tag penetration test the specimens were fixed in plasticine 1×1 mm. A mould with a diameter of 2 mm was glued over the specimen in the pulp chamber using plasticine." - What was the exact size? 20mm is the length? Please clarify?
- Specify more details on the specimen preparation particularly for the CLSM, the specimen should be different than for the shear bond test.
- The statistical method written in the abstract is different than in the method section, was there any t test performed (as stated in abstract)?

**Results**

- "Table 1 shows that the longest penetration of resin tag is in the 35% GT group with a mean value of 190.1 (SD 33.6) μm. Meanwhile, the post-bleaching dentin group showed the shortest resin tag penetration with a mean value of 87.4 (SD 6.9) μm. **^30^** The results of the one-way ANOVA test showed a value of 0.001 (p<0.05), which means that there was a significant difference in the depth of penetration of the resin tags from the five groups." - What does the citation for 30 mean? No citations should be included in the results section.
- Please change the writing in Table 1 to a more suitable scientific table. Since all sample numbers for the resin penetration are the same in each group (n=6) and also for the shear bond strength (n=5), there is no need to include a column sample number. Instead, mention the measurement's unit (m) and clarify in the Figure legend what the x ,y, z, superscript mean?
- How was the CLSM quantitatively evaluated, and how did the author determine the mean value of each specimen's penetration depth?
- The image of CLSM is unclear, there is no significant adhesive penetration seen, and the image of CLSM is vague. Some adhesive fragments from the dentin cuttings might be mistakenly interpret as adhesive penetration. There is no continuous adhesive penetration from the interface to the tubules seen, therefore it might not be reliable to quantitative measurement. How thick were the samples, in case the specimen was too thick?
- Images needs to be improved, and clearly state the what the star, rectangle, red and blue colour represent.

Is the work clearly and accurately presented and does it cite the current literature?

Partly

If applicable, is the statistical analysis and its interpretation appropriate?

Partly

Are all the source data underlying the results available to ensure full reproducibility?

Partly

Is the study design appropriate and is the work technically sound?

Partly

Are the conclusions drawn adequately supported by the results?

Yes

Are sufficient details of methods and analysis provided to allow replication by others?

Partly

Reviewer Expertise:

Endodontics, oral microbiology, dental materials

I confirm that I have read this submission and believe that I have an appropriate level of expertise to confirm that it is of an acceptable scientific standard, however I have significant reservations, as outlined above.

# Response

**Published:** 2023-08-24

**Authors:** Paramita Widyandari

**Affiliations:** Conservative Dentistry, Universitas Indonesia, Depok, West Java, Indonesia

We would like to thank the reviewers for their thoughtful comments on our manuscript. We have carefully considered their suggestions in revising our manuscript, and we believe that the revised manuscript is improved and suitable for publication. Detailed responses to the comments are presented in this document.

**Abstract**

 1. Please mention the aim of the study in the abstract.

***Response:***

*We have revised the manuscript and you can find the aim of the study in background section in abstract line 3-5*

 “The aim of this study is to evaluate the effect of 10% and 35% GT extract after 2 minutes of application on dentin shear bond strength and resin tags penetration depth after non-vital bleaching *”*

 2. Please specify the method in abstract section including the statistics. What was the post hoc analysis following the ANOVA and what was the t test for? (there was no t-test mention in the script).

***Response:***

*The data was analyzed using one-way ANOVA followed by post-hoc Tamhane test for resin tag penetration. Then, we used post-hoc Bonferroni test for shear bond strength test. However, there is no t-test we did, which was accidentally mistaken in the original manuscript. We revise it as mention in paragraph 10 line 1-3*

*“*The data was analyzed using one-way ANOVA, followed by post-hoc Tamhane test for resin tag penetration and post-hoc Bonferroni test for shear bond strength test (IBM SPSS Statistics 24 version program, IBM Corp., Armonk, NY, USA). *”*

 3. Please change 'aquabidest' to 'distilled water'.

***Response:***

*This issue has already been addressed in the abstract part of the original manuscript:*

*“*Soon after, 10% and 35% GT extract gel were applied on dentin for 2 minutes, then the specimens were rinsed-off with distilled water for 2 minutes and dried. *”*

 4. Results is unclear, it seemed that author only explained comparison between 2 groups (leads to assumption the result of t test, but then it was not in the methods and results of the manuscript).

***Response:***

*We agree with the reviewer. However, due to our focus is on the effect of green tea extract antioxidant application on dentin shear bond strength and resin tag penetration depth after non-vital bleaching dentin, therefore, our conclusion is focusing on the antioxidant materials only. The other groups are positive and negative control groups which were already mentioned in discussion section, paragraph 4 line 2-4.*

 “From the post-hoc test, it appears that there is a significant difference in the resin tag penetration and shear bond strength between the 35% GT group and the post-bleaching dentin group (p<0.05). *”*

 5. Please specify the results of shear bond strength of the 5 groups, then mention the significance. Since author is not only comparing 10% and 35% GT extract, how was the results for the other groups tested (highest? Lowest?)? Was there any host hoc analysis done?

***Response:***

*Thank you for the question and we agree with the reviewer. Unfortunately, due to the limitation number of the words in the abstract it cannot be written in the abstract. Therefore, we mentioned it on the results part, paragraph 4, line 2-7.*

*“*The results show a significant difference in the shear bond strength between the 10% GT group and the 35% GT group (p=0.001). The 35% GT group showed the higher shear bond strength compared to 10% GT group. These results show that the application of 35% GT extract can increase the post-bleaching shear bond strength more than the application of 10% GT. The 10% GT group showed lower shear bond strength than normal dentin. There was no significant difference of shear bond strength between the 35% GT group and the normal dentin group. *”*

**Introduction**

 1. What was the rationale in using 10 and 35% extract? Generalizing the results from 35% sodium ascorbate to this study is irrelevant. GT extract differs from sodium ascorbate; therefore, it cannot be used as a standard for concentration.

***Response:***

*The rationale in using 10% GT extract has been conducted by Alhasani et al. (*Al-Hassani AA, Al-Shamma A: Effect of delayed bonding and different antioxidants on composite restoration microleakage of internally bleached teeth. *Adv. Dent. Oral Health.* 2018;9:001–006. 10.19080/ADOH.2018.09.555762 *) and we it was mentioned in the reference section number 10. Moreover, the background in using 10% GT extract already written in the original manuscript, paragraph 3 line 1-4.*

*“Applying of 10% green tea (10% GT) extract for 10 minutes effectively increased shear bond strength and reduced microleakage formation, which is closely related to the ability of the resin tag penetration which can affect the retention of the composite resin restoration to dentin”.*

*The rationale in using 35% GT was based on the statement that the concentration of antioxidants must be proportional to the concentration of hydrogen peroxide (*The concentration of antioxidants must be proportional to the concentration of hydrogen peroxide, and the application of 35% sodium ascorbate for 2 minutes has been proven to remove free radicals after the application of 35% H _2_O _2_. *, Reference number 13: *Freire A, Souza EM, de Menezes Caldas DB , et al.: Reaction kinetics of sodium ascorbate and dental bleaching gel. *J. Dent.* 2009;37(12):932–936. 19660514 10.1016/j.jdent.2009.07.008 *). As we used 35% hydrogen peroxide in this study, thus, the authors choosing 35% green tea extract for other group.*

 2. Introduction paragraph 3:“The concentration of antioxidants must be proportional to the concentration of hydrogen peroxide, and **the application of 35% sodium ascorbate for 2 minutes has been proven to remove free radicals after the application of 35% H2O _2_.** ^13^ Therefore, 35% green tea (35% GT) extract was used in this study, and it was expected that it could remove free radical residue and increase the resin tag penetration depth. ^13^ **The application of 35% sodium ascorbate for 2 minutes has previously been shown to be effective in removing residual free radicals after application of 35% H2O _2_**. ^14”^ - Even though they have different citations, the two phrases have the same meaning. Avoid overwriting it.

***Response:***

 Thank you for your comment. *This issue has already been addressed in the introduction part* *in the paragraph 3 line 3-5.*

*“*Applying of 10% green tea (10% GT) extract for 10 minutes effectively increased shear bond strength and reduced microleakage formation, which is closely related to the ability of the resin tag penetration which can affect the retention of the composite resin restoration to dentin. ^10^^ –^^12^ The concentration of antioxidants must be proportional to the concentration of hydrogen peroxide, and the application of 35% sodium ascorbate for 2 minutes has been proven to remove free radicals after the application of 35% H _2_O _2_. ^13^[PW1] Therefore, 35% green tea (35% GT) extract was used in this study, and it was expected that it could remove free radical residue and increase the resin tag penetration depth. ^13^ *”*

 3. What was the colour of the green tea extract? Isn’t there a chance of staining to the tooth compromising the bleaching results?

***Response:***

*The color of the green tea extract is dark green using colorimetry test, below are the results and no significant discoloration were found.*

*Result of Colorimetri test*

*Number of samples*

*Before application of 35% GT for 2 minutes*

*After Application of 35% GT for 2 minutes*

*1*

*65.835*

*67.336*

*2*

*60.672*

*61.419*

*3*

*54.489*

*58.709*

T-Test [DataSet3]

**Methods**

 1. "The dentin surfaces of the specimens were flattened using 600- and 1200-grit sandpaper and polished with felt discs (Arotec, Cotia, SP, Brazil) impregnated with alumina paste (0.5 μm)." - what was the final specimen dimension?

***Response:***

*We checked the dimension of the samples after the polishing step, and the final specimen thickness is 3 mm and if the final thickness is decreased more than 0.5 mm, specimens will discarded and replaced.*

 2. "The specimens were washed ultrasonically in distilled water for 5 minutes to eliminate any residue. For the shear bond strength test the **specimens were fixed in self-cure acrylic 20 mm**, and for the resin tag penetration test the specimens were fixed in plasticine 1×1 mm. A mould with a diameter of 2 mm was glued over the specimen in the pulp chamber using plasticine." - What was the exact size? 20mm is the length? Please clarify?

***Response:***

*The dimensions of the self-cure acrylic for the placement of the specimen were both 20 mm in diameter (cylindrical form) and in the length.*

 3. Specify more details on the specimen preparation particularly for the CLSM, the specimen should be different than for the shear bond test.

***Response:***

*The specimen preparation procedures are same for CLSM and shear bond test. Additionally, there is only one difference in the specimen placement media (CLSM is using plasticine and shear bond strength is using self-curing acrylic). The procedures detailed and written in the manuscript in methods section, paragraph 2, line 1-6.*

*“*The dentin surfaces of the specimens were flattened using 600- and 1200-grit sandpaper and polished with felt discs (Arotec, Cotia, SP, Brazil) impregnated with alumina paste (0.5 μm). The specimens were washed ultrasonically in distilled water for 5 minutes to eliminate any residue. For the shear bond strength test the specimens were fixed in self-cure acrylic 20 mm, and for the resin tag penetration test the specimens were fixed in plasticine 1×1 mm. A mould with a diameter of 2 mm was glued over the specimen in the pulp chamber using plasticine.

*”*

 4. The statistical method written in the abstract is different than in the method section, was there any t-test performed (as stated in abstract)?

***Response:***

*Thank you for the comment, and we apologize for the accidentally mistaken. We did not use t-test in this study and we wrote it in methods section (paragraph 10 line 1-3).*

 “The data was analyzed using one-way ANOVA, followed by post-hoc Tamhane test for resin tag penetration and post-hoc Bonferroni test for shear bond strength test (IBM SPSS Statistics 24 version program, IBM Corp., Armonk, NY, USA). *”*

**Results**

 1. "Table 1 shows that the longest penetration of resin tag is in the 35% GT group with a mean value of 190.1 (SD 33.6) μm. Meanwhile, the post-bleaching dentin group showed the shortest resin tag penetration with a mean value of 87.4 (SD 6.9) μm. **^30^** The results of the one-way ANOVA test showed a value of 0.001 (p<0.05), which means that there was a significant difference in the depth of penetration of the resin tags from the five groups." - What does the citation for 30 mean? No citations should be included in the results section.

***Response:***

*Thank you for the comment, and we apologize for the accidentally mistaken. We revised it.*

*“*Table 1 shows that the longest penetration of resin tag is in the 35% GT group with a mean value of 190.1 (SD 33.6) μm. Meanwhile, the post-bleaching dentin group showed the shortest resin tag penetration with a mean value of 87.4 (SD 6.9) μm. *”*

 2. Please change the writing in Table 1 to a more suitable scientific table. Since all sample numbers for the resin penetration are the same in each group (n=6) and also for the shear bond strength (n=5), there is no need to include a column sample number. Instead, mention the measurement's unit (m) and clarify in the Figure legend what the x ,y, z, superscript mean?

***Response:***

 Thank you for your comment. We revise it as detailed in the manuscript page 6-7

**Table 1.** Mean, standard deviation, and significancy between group of resin tag penetration (µm)

**Treatment**

**Number of specimens**

**Mean (SD)**

**p-value**

 Normal dentin

 6

 146.9 (10.1) *^x^*

0.001*

 Post-bleaching dentin

 6

 87.4 (6.9) *^y^*

 Delay 2 weeks

 6

 134.4 (9.5)

 10% GT

 6

 107.2 (18.9) *^x,z^*

 35% GT

 6

 190.1(33.6) ^y,z^

 GT = Green Tea;  x= significance difference normal dentin with 10% GT; y= significance difference post bleaching dentin with 35% GT; z = significance difference 10%GT with 35% GT

 * One-way ANOVA test with p<0.05

**Tabl** **e 2.** Mean, standard deviation, and significancy between group of shear bond strength (MPa)

**Treament group**

**Number of specimens**

**Mean** **(SD)**

p-value*

 Normal dentin

 5

 10.14 (1.85) ^a^

0.001*

 Post-bleaching dentin

 5

**3.52 (0.22)** **^b^**

 Delay 2 weeks

 5

 8.65 (2.09)

 10% GT

 5

 5.42 (0.54) ^a,c^

 35% GT

 5

1.
  1. **2.68)** **^b,c^**

GT = Green Tea; a= significance difference normal dentin with 10% GT; b= sigficance difference post bleaching dentin with 35% GT ; c= significance difference 10% GT with 35% GT

 *One-way Anova test with p<0.05

 3. How was the CLSM quantitatively evaluated, and how did the author determine the mean value of each specimen's penetration depth?

***Response:***

 We measure the continuous and longest penetration depth from each specimen based on the previous studies “ The SEM observation of resin inltration samples showed resin-inltrated tags. InIIa, the deepest depths of inltration were determined to range from 245 to 317 µm and

 the mean depth was 284 µm”(Effect of Resin Inltration on Bleached Enamel: An In Vitro Evaluation of Micromorphology, Resin Penetration and Microhardness, Zhen Wei, et al , GuangzhouInstitute of Oral Disease). 

Table 3

 4. The image of CLSM is unclear, there is no significant adhesive penetration seen, and the image of CLSM is vague. Some adhesive fragments from the dentin cuttings might be mistakenly interpret as adhesive penetration. There is no continuous adhesive penetration from the interface to the tubules seen, therefore it might not be reliable to quantitative measurement. How thick were the samples, in case the specimen was too thick?

***Response:***

*We revise it and please find it in the results section, page 6 and here.*

 Regarding the thickness of the samples, it written on the manuscript methods section, page 3, paragraph 2 line 1-3

 “The dentin surfaces of the specimens were flattened using 600- and 1200-grit sandpaper and polished with felt discs (Arotec, Cotia, SP, Brazil) impregnated with alumina paste (0.5 μm) until a specimen thickness of 3 mm was obtained”

 5. Images needs to be improved, and clearly state the what the star, rectangle, red and blue colour represent.

***Response:***

 Thank you for your comment. We revise it as detailed in the manuscript results section, page 7.

 Figure 1. The red arrow indicates the depth of penetration of the resin tag on (A) Normal dentin group, (B) Post bleaching group, (C) Delay 2 weeks group, (D) 10% GT group, (E) 35% GT group. Rectangle indicates the adhesive; star indicates the hybrid layer; blue area means there is no adhesive penetration/no fluorescence, red area means the thickest fluorescence.
